# A digital tool for prevention and management of cold weather injuries—Cold Weather Ensemble Decision Aid (CoWEDA)

**DOI:** 10.1007/s00484-021-02113-0

**Published:** 2021-04-04

**Authors:** Xiaojiang Xu, Timothy P. Rioux, Julio Gonzalez, Eric O. Hansen, John W. Castellani, William R. Santee, Anthony J. Karis, Adam W. Potter

**Affiliations:** 1 Biophysics and Biomedical Modeling Division, U.S. Army Research Institute of Environmental Medicine, 10 General Greene Avenue, Natick, MA 01760-5007 USA; 2 Oak Ridge Institute for Science and Education (ORISE), Oak Ridge, TN USA; 3 Thermal and Mountain Medicine Division, U.S. Army Research Institute of Environmental Medicine, Natick, MA USA; 4 Military Nutrition Division, U.S. Army Research Institute of Environmental Medicine, Natick, MA USA

**Keywords:** Clothing, Frostbite, Hypothermia, Thermoregulation model, Manikin

## Abstract

This paper describes a Cold Weather Ensemble Decision Aid (CoWEDA) that provides guidance for cold weather injury prevention, mission planning, and clothing selection. CoWEDA incorporates current science from the disciplines of physiology, meteorology, clothing, and computer modeling. The thermal performance of a cold weather ensemble is defined by endurance times, which are the time intervals from initial exposure until the safety limits are reached. These safety limits correspond to conservative temperature thresholds that provide a warning of the approaching onset of frostbite and/or hypothermia. A validated six-cylinder thermoregulatory model is used to predict human thermal responses to cold while wearing different ensembles. The performance metrics, model, and a database of clothing properties were integrated into a user-friendly software application. CoWEDA is the first tool that allows users to build their own ensembles from the clothing menu (i.e., jackets, footwear, and accessories) for each body region (i.e., head, torso, lower body, hands, feet) and view their selections in the context of physiological strain and the operational consequences. Comparison of predicted values to skin and core temperatures, measured during 17 cold exposures ranging from 0 to −40°C, indicated that the accuracy of CoWEDA prediction is acceptable, and most predictions are within measured mean ± SD. CoWEDA predicts the risk of frostbite and hypothermia and ensures that a selected clothing ensemble is appropriate for expected weather conditions and activities. CoWEDA represents a significant enhancement of required clothing insulation (IREQ, ISO 11079) and wind chill index-based guidance for cold weather safety and survival.

## Introduction

Cold weather is a persistent danger during outdoor activities (Imray and Oakley 2005). Cold stress has deleterious effects on health, performance, and military readiness and may eventually lead to cold injuries, e.g., frostbite and life-threatening hypothermia (Castellani and Tipton 2015; Holmer 2009; DeGroot et al. 2003; Imray and Oakley 2005). Despite advancements in personal protective equipment, cold injuries continue to affect active military and civilian personnel. From 2012 through 2017, a total of 2717 US service members had at least one medical encounter for a cold injury with a primary diagnosis of frostbite as the most common (O'Donnell et al. 2017). It was reported that 21% of Canadian soldiers developed frostbite during a field training exercise at air temperatures of −21°C and a wind chill index of −44°C (Sullivan-Kwantes et al. 2017). The hands and feet were the most susceptible, followed by the face and ears (Sullivan-Kwantes et al. 2017; Sullivan-Kwantes and Goodman 2017). Civilian populations are also at risk, most often in outdoor workers and winter sports participants (Makinen and Hassi 2009; Heil et al. 2016). Wearing cold weather clothing is the primary way to reduce cold injury (Heil et al. 2016) and is expected to provide protection against both extremity cold injuries and hypothermia. Thus, selection of adequate cold weather ensembles is a vital mitigation strategy for preventing cold injury.

Human performance is degraded and cold injury risk increases when body temperature drops. Hypothermia occurs when the body core temperature falls below 36°C. When the skin temperature of an extremity begins to fall, it causes discomfort, pain, numbness, performance deterioration, and eventually local cold injury. Hand manual performance deteriorates as hand skin temperatures decrease (Geng 2001; Heus et al. 1995). Cold feet affect balance and walking and may increase the risk of slipping (Holmer 2009). The cold injury risk increases significantly when skin temperatures drop below 5°C (Kuklane 2009; Heus et al. 1995; Castellani et al. 2006).

At present, methods of cold weather ensemble evaluation are based on simple whole-body heat balance equations. One popular method is the required clothing insulation (IREQ), which is incorporated into the International Standard ISO 11079 (Holmer 1984, 1988; ISO 11079 2007; Aptel 1988; d'Ambrosio Alfano et al. 2013; Besnard et al. 2004; Gao et al. 2015). ISO 11079 determines the insulation of cold weather ensembles required to maintain heat balance for different sets of environmental conditions and work intensity and provides limited guidance for the evaluation of extremity cooling. Another standard, ASTM F2732-16, predicts the temperature rating for comfort at two activity levels using simple whole-body heat loss models (ASTM International 2016a). However, ISO 11079 and ASTM F2732-16 do not provide any information on required insulation for extremity (hands and feet) protection**.** Furthermore, the primary output for IREQ and ASTM F2732-16 are clothing insulation values (thermal resistance) and temperature values, respectively. These values are difficult for standard users to interpret and translate into sensible decisions in terms of clothing selection, safe exposure duration, or the potential risks of cold injury. Therefore, the two existing methods of cold weather ensemble evaluation, ISO 11079 and ASTM F2732-16, are limited and provide incomplete guidance for determining clothing items and preventing cold injuries.

Whether for daily life or during recreational, occupational, or military activities in the cold, it is critical to know how long a specific ensemble will allow users to function or work safely under a given set of environmental conditions or which ensembles are most suitable for specific conditions and tasks. The objective of our work is to develop software that addresses all of the complicated requirements needed for cold protection and specifically to quantify the thermal performance of cold weather ensembles. Our work includes (1) development of a human-centric approach to quantify thermal performance of cold weather ensembles using physiological criteria or safety limits; (2) development of a capability to translate ensemble thermal properties into meaningful guidance for safe operational limits using thermoregulatory modeling; and (3) development of a user-friendly software application for operation on mobile and desktop platforms, the Cold Weather Ensemble Decision Aid (CoWEDA), to enable end users use of this tool in operational settings.

## Methods

### System design

**Fig. 1:**
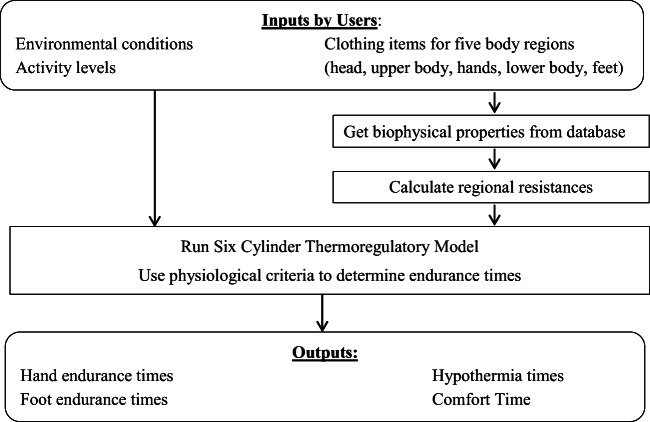
Cold Weather Ensemble Decision Aid flowchart

CoWEDA integrates physiological safety criteria, a six cylinder thermoregulatory model (SCTM), a database of clothing biophysical properties, and algorithms for calculating regional thermal and evaporative resistances of a selected ensemble into a user-friendly software application. Figure 1 shows a software flow chart for CoWEDA. The environmental conditions, activity level, and the regional thermal and evaporative resistances of the ensemble are SCTM inputs. The database of biophysical properties includes intrinsic thermal and evaporative resistance values for individual clothing items. The regional thermal and evaporative resistances of ensembles are calculated from the values of the selected individual clothing items

### Performance metrics of cold weather ensembles

The role of cold weather ensembles is to maintain skin and core temperatures and prevent them from dropping below critical levels. Accordingly, thermal performance of cold weather ensembles were defined as endurance times rather than thermal insulation to represent the protection level an ensemble provides. Endurance times are defined as the duration until these physiological parameters (i.e., core temperature, toe and finger skin temperatures) drop to a low level, i.e., physiological threshold, indicating a high risk of cold injuries. Thus, the metrics focus on the safety or performance of the person and quantifies the protection level needed by a cold weather ensemble to maintain safe temperatures. Based on the published values in the literatures (ISO 11079 2007; Gonzalez et al. 1998; Gao et al. 2015; Heus et al. 1995; Kuklane 2009; Holmer 2009; Castellani et al. 2006; ASHRAE 2013), the physiological criteria in Table 1 were selected to provide guidance for the prevention of cold injury. These criteria were used to determine endurance times and to quantify the thermal performance metrics of cold weather ensembles. During exercise in the cold, the sweat accumulating in the clothing may reduce the insulation (i.e., protection level) and thus increase the risk of cold injury. The inclusion of skin wettedness is to ensure that the ensemble is appropriate to the exercise intensity and to avoid excessive sweating or overheating.

**Table 1:** Physiological criteria for cold injury prevention and for thermal performance metrics of cold weather ensembles

Thermal performance metrics	Physiological parameters	Criteria	Signs and symptoms
Functional time	Core temperature	36°C	Mild hypothermia
Hand endurance time	Finger skin temperature	5°C	Extreme pain or numbness Significant impairment in manual dexterity
Foot endurance time	Toe skin temperature	5°C	Extreme pain or numbness
Exposed skin endurance time	Exposed skin temperature	5°C	Extreme pain or numbness
Comfort time	Skin wettedness	0.5	Discomfort, skin and underwear getting wet

### Database of clothing properties

A database was created to collect clothing properties, e.g., thermal resistance, evaporative resistance, and images. The clothing items included in the database are components of the US Army Generation III Extended Cold Weather Clothing System (GEN III ECWCS) which includes more than 50 items, including clothing, headgear, handwear, and footwear. Table 2 presents an example from the database, which include regional thermal and evaporative resistances of 13 individual clothing items. The regions included were the head, torso, arm, hand, leg, and foot. The regional data makes it possible to quantify the contributions of individual clothing items to the overall protection provided by the ensemble and to evaluate local thermal performance of accessories such as gloves and boots.

**Table 2:** Intrinsic thermal and evaporative resistances of individual clothing items

Item name	Thermal resistance (m^2^°C/W)						Evaporative resistance (m^2^Pa/W)					
	Head	Torso	Arm	Hand	Leg	Foot	Head	Torso	Arm	Hand	Leg	Foot
Gen III ECWCS lightweight cold weather undershirt	0.000	0.086	0.064	0.007	0.002	0.000	0.00	9.86	6.52	0.00	1.02	0.00
Gen III ECWCS lightweight cold weather drawers	0.000	0.022	0.000	0.000	0.052	0.000	0.00	3.31	0.00	0.00	6.81	0.00
Gen III ECWCS midweight cold weather shirt	0.004	0.107	0.105	0.013	0.000	0.000	0.32	12.96	13.33	0.00	0.00	0.00
Gen III ECWCS midweight cold weather drawers	0.000	0.026	0.000	0.000	0.092	0.000	0.00	3.41	0.00	0.00	10.08	0.00
Gen III ECWCS fleece cold weather jacket	0.008	0.163	0.204	0.000	0.001	0.000	0.47	14.20	21.70	0.00	0.03	0.00
Gen III ECWCS cold weather wind jacket	0.000	0.109	0.085	0.000	0.001	0.000	0.00	13.63	16.27	0.00	0.00	0.00
Gen III ECWCS soft shell jacket	0.005	0.147	0.101	0.000	0.001	0.000	0.09	46.13	55.94	0.00	0.00	0.00
Gen III ECWCS soft shell trouser	0.000	0.045	0.000	0.000	0.117	0.000	0.00	8.06	0.00	0.00	70.57	0.00
Gen III ECWCS cold wet weather jacket	0.003	0.136	0.107	0.000	0.001	0.000	0.00	34.25	31.81	0.00	0.00	0.00
Gen III ECWCS cold wet weather trouser	0.000	0.031	0.000	0.000	0.109	0.000	0.00	5.58	0.00	0.00	39.38	0.00
Gen III ECWCS extreme cold weather parka (stowed hood)	0.000	0.572	0.456	0.000	0.002	0.000	0.00	65.91	65.41	0.00	0.00	0.00
Gen III ECWCS extreme cold weather trouser	0.000	0.052	0.000	0.000	0.387	0.000	0.00	7.41	0.00	0.00	69.06	0.00
Gen III ECWCS extreme cold weather parka (freed hood)	0.141	0.572	0.456	0.000	0.002	0.000	29.87	65.91	65.41	0.00	0.00	0.00

A paradigm of testing all individual layers of a multi-layer clothing system was developed and used to test all components and collect thermal and evaporative resistance values for the database (Rioux et al. 2018; Potter et al. 2018). The thermal and evaporative resistances of clothing were measured with sweating thermal manikins, i.e., whole-body, hand, foot, and head thermal manikins (Thermetrics, Seattle, WA; www.Thermetrics.com). All manikin testing was conducted according to ASTM International standards F1291-16 and F2370-16 (ASTM International 2016b, c). Thermal resistance was measured at an environment of 20°C, 50%RH, and 0.4 m·s^−1^. Evaporative resistance was measured at an environment of 35°C, 40%RH, and 0.4 m·s^−1^. The data were processed and saved in the database as shown in Table 2. Thermal and evaporative resistances for SCTM inputs were calculated from the value of individual items using newly developed algorithms. Details of the measurement and processing procedures were reported in separated papers (Rioux et al. 2018; Potter et al. 2018; Rioux et al. 2020).

### Thermoregulatory Model

**Fig. 2:**
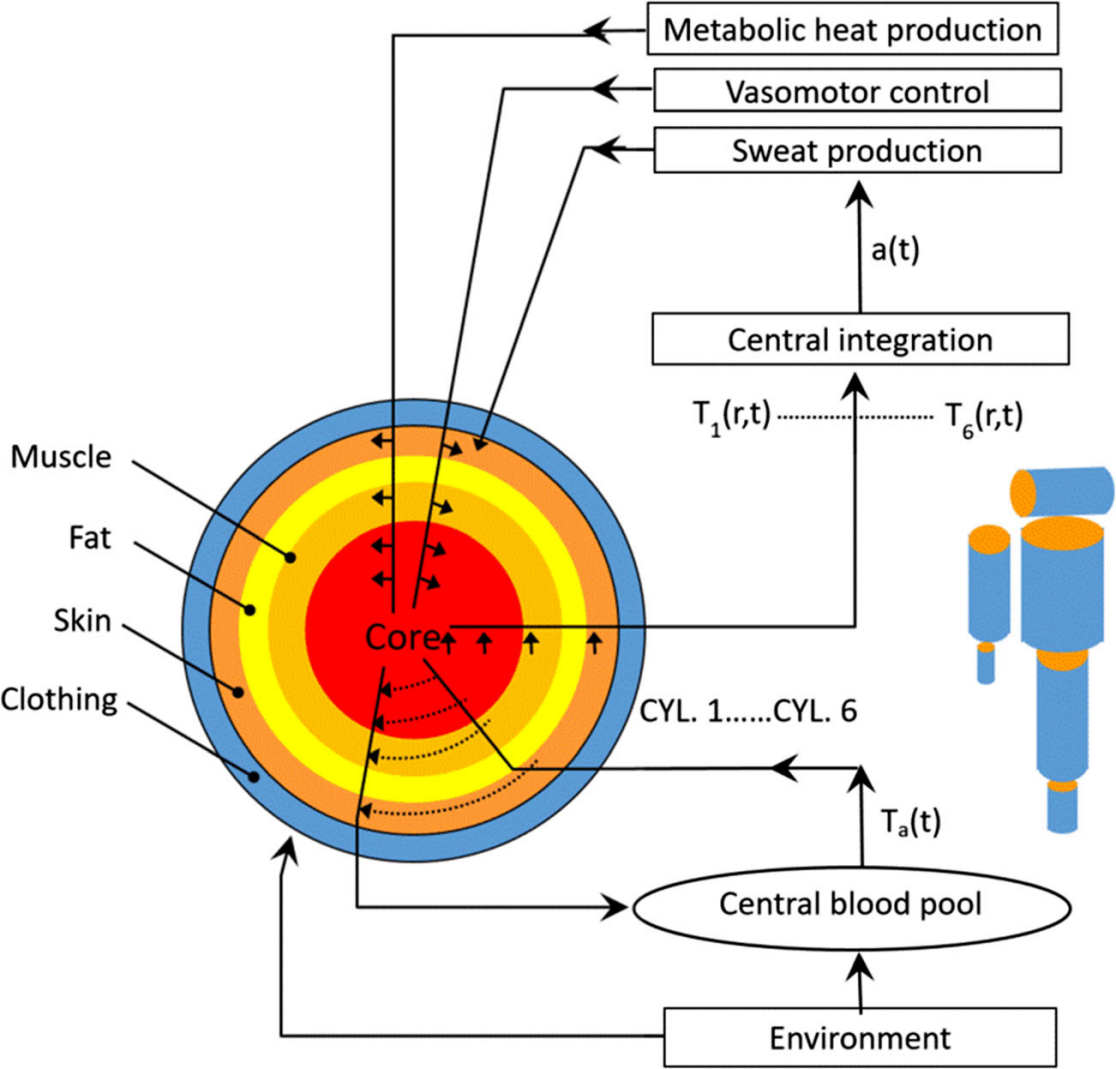
Schematic of the six cylinder thermoregulatory model (SCTM)

The SCTM is used to predict physiological responses to determine the endurance times. It is a rational model and is based on first principles of physiology and the physical laws of heat transfer (Xu and Werner 1997; Xu et al. 2005b). As shown in Fig. 2, the human body is subdivided into six segments representing the head, torso, arms, legs, hands, and feet. Each segment is further divided into concentric compartments representing the core, muscle, fat, and skin. The integrated thermal signal to the thermoregulatory controller is composed of the weighted thermal input from thermal receptors at various sites distributed throughout the body. The difference between this signal and its threshold activates the thermoregulatory actions: shivering heat production, vasodilation/vasoconstriction, and sweat production.

The SCTM has been validated for a broad range of conditions, including heat, cold, and water immersion (Xu and Werner 1997; Xu et al. 2005b; Castellani et al. 2007; Xu et al. 2007; Xu and Santee 2011; Xu et al. 2011). SCTM inputs include individual characteristics, intensity of activity, environmental conditions, and clothing properties (i.e., thermal resistance and evaporative resistance) for each of the six body regions. SCTM predicts physiological responses, e.g., core temperatures, skin temperatures and sweat rates for six body regions.

### Cold Weather Ensemble Decision Aid

**Fig. 3:**
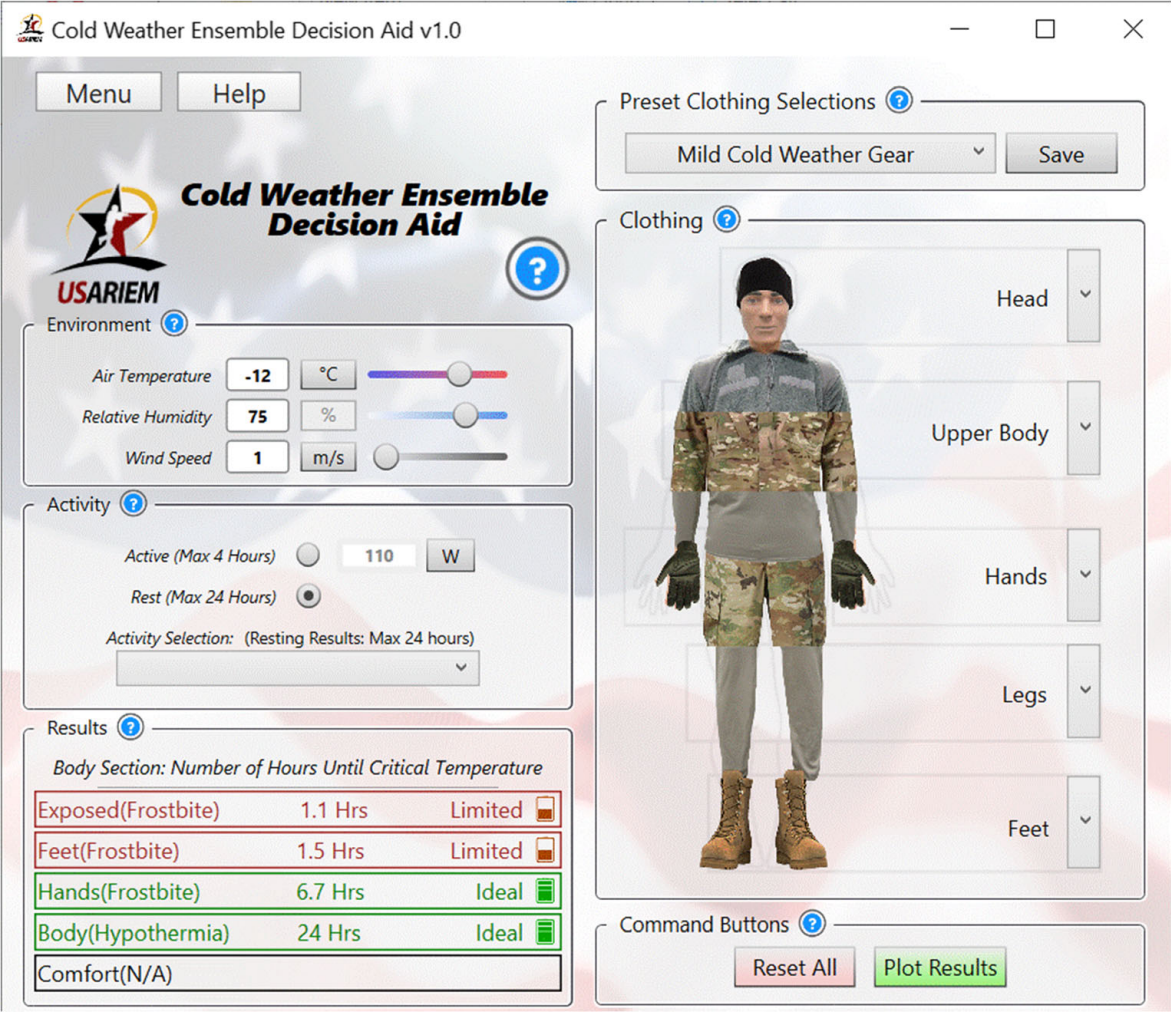
Cold Weather Ensemble Decision Aid (CoWEDA)

As shown in Fig. 3, CoWEDA is a user friendly and guides selection of the most appropriate cold weather ensemble to safely execute the anticipated mission physical activities in forecasted cold weather. Users enter or select environmental conditions, input activity or work intensity from a menu, and select clothing items using a pull-down menu. Then CoWEDA calculates and displays the results as follows:

1. Endurance time of the uncovered body surface areas (e.g., face; the hands, if no handwear is worn) is the time for the temperature of the skin exposed to air to reach 5°C; below this threshold, the probability of surface cold injury increases significantly.
2. Hand endurance time is the time for the predicted hand skin temperature to reach 5°C, indicating that a cold injury is likely to occur soon; below this threshold, the probability of frostbite increases significantly.
3. Foot endurance time is the time for the predicted foot skin temperature to reach 5°C, indicating that a cold injury is likely to occur soon; below this threshold, the probability of frostbite increases significantly.
4. Body endurance time is the time until the predicted core temperature reaches 36°C, indicating there is an increased risk of hypothermia if the core temperature continues to decrease.
5. Comfort time is the time until the predicted skin wettedness (ω) reaches 0.5, indicating that underwear starts to get uncomfortably wet and a reduction in clothing insulation can be expected.

**Fig. 4:**
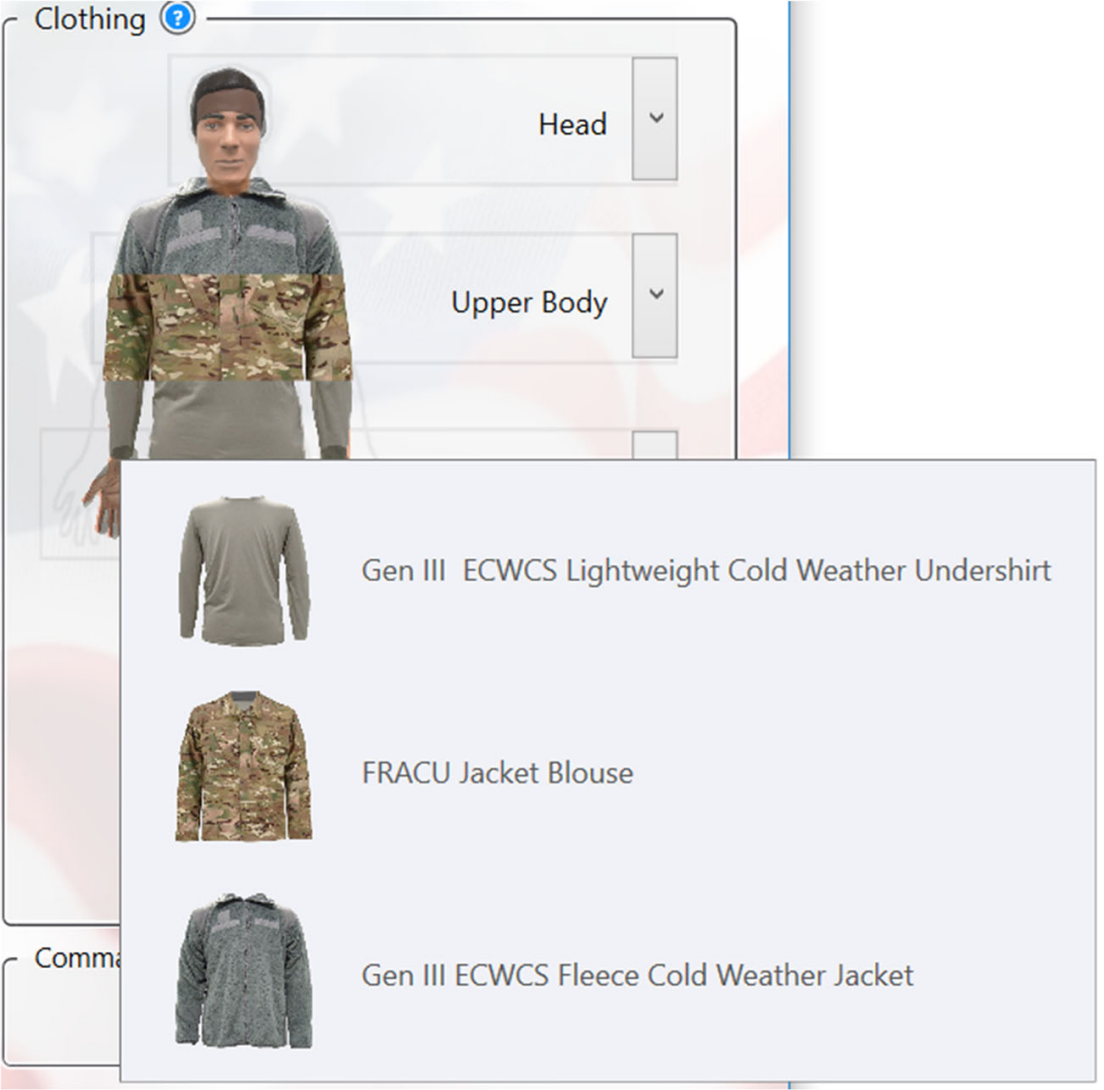
Selected clothing for the region, e.g., upper body

**Fig. 5:**
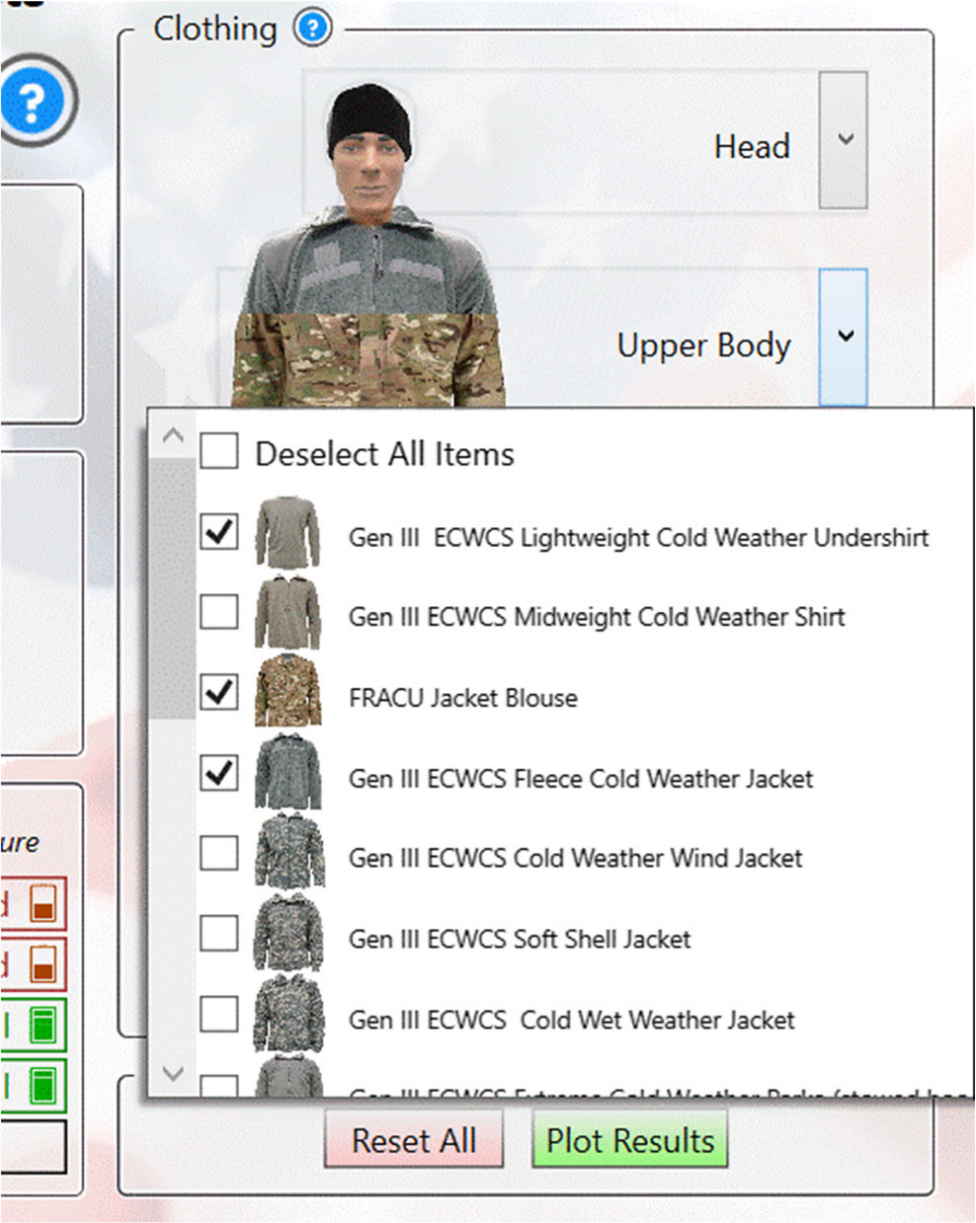
Menu of clothing options for each body region, e.g., upper body

**Fig. 6:**
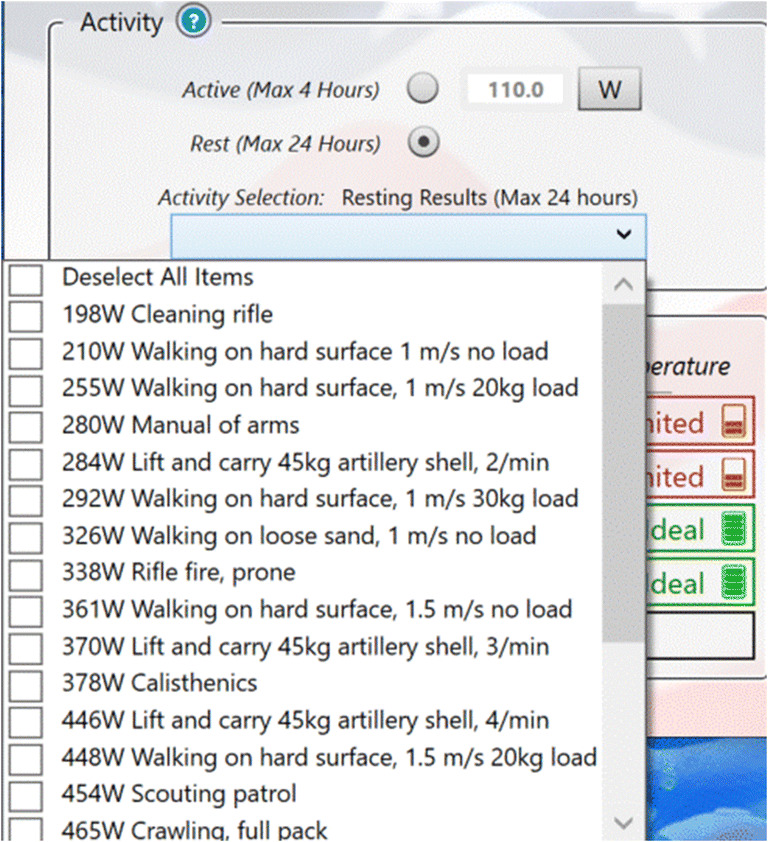
Activity panel pull down menu

CoWEDA was designed to be simple, clear, and user-friendly. The clothing selection panel provides options to select clothing for five body regions: the head, upper body, hand, lower body, and foot, as shown in Fig. 2. When the cursor is hovered over the body zone, it shows the selected clothing for the zone, as shown in Fig. 4. When clicking the dropdown icon “v” on the right, the panel displays the menu of clothing options for each zone, as shown in Fig. 5. Activities for common tasks can be selected through a pull down menu, as shown in Fig. 6.

**Fig. 7:**
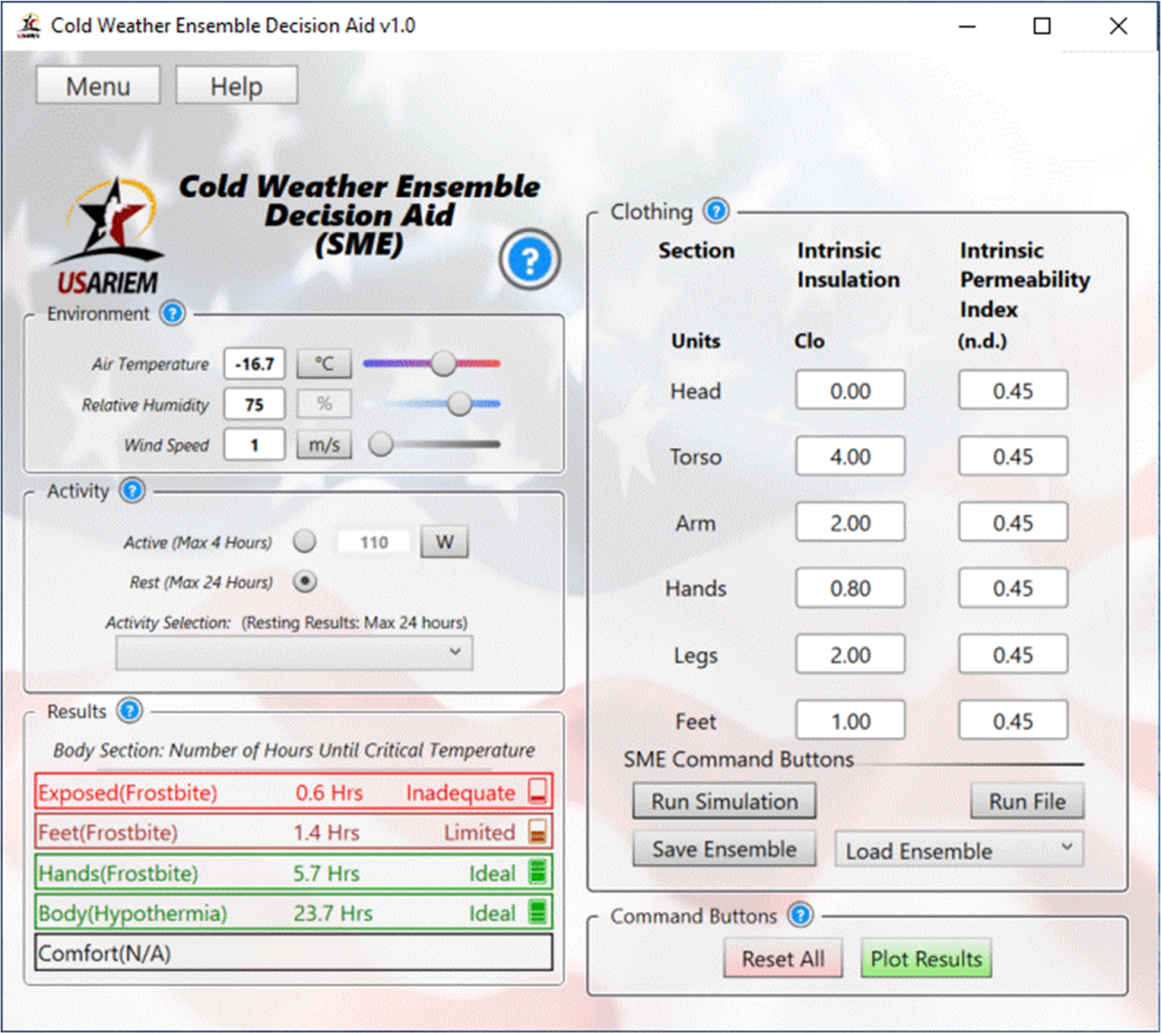
Cold weather ensemble Decision Aid For Subject Matter Expert (CoWEDA-SME)

Figure 7 shows a version of CoWEDA for a subject matter expert (CoWEDA-SME). Users need to input thermal and evaporative resistances for six body regions: the head, torso, arms, legs, feet, and hands. This version assists technically oriented users, such as clothing designers, material developers, and researchers, to evaluate the performance of cold weather ensembles, to determine specifications for new ensembles, and to assess if an ensemble meets mission requirements.

### Data analysis

Differences between observed temperatures and CoWEDA predictions were evaluated by comparing the root mean square deviation (RMSD) of each trial with the observed standard deviation (Haslam and Parsons 1994). This statistic is used to quantitatively determine the goodness of fit between model predictions and observed data. The RMSD (°C) is defined as

$$ \mathrm{RMSD}=\sqrt{\frac{1}{n}\sum \limits_{i=1}^n{d}_i^2} $$

where *d*_i_ is the difference between observed and predicted core temperature response at each time point (°C) and *n* is the number of time points examined with an interval of 10-min used. The prediction was considered valid if the RMSD fell within the SD of the observed values (Gonzalez et al. 1997; Castellani et al. 2007; Xu et al. 2005b).

## Results—validation with human studies

### Physiological data

Data from the following three human studies were used to validate the CoWEDA. In study 1, six fit males rested (*M* = 71 W·m^−2^) and did treadmill exercise (*M* = 171 W·m^−2^) in cold air environments of 0°C, −20°C, and −30°C; RH 20–25%; and air velocity 1.34 m·s^−1^ for a maximum of 120 min (Gonzalez et al. 1989). They wore the original Extended Cold Weather Clothing System (ECWCS I) with three different gloves: the light duty glove, the heavy duty glove, and the Arctic mitten. The ECWCS ensemble (insulation, 3.6 clo; weight, 10.1 kg) consisted of skin-tight polypropylene underwear, polyester/cotton fatigues, polyester-insulated liners, balaclava, vapor-barrier boots, and polytetrafluorethylene-lined outer garments plus handwear. Rectal (*T*_core_), middle finger (*T*_mf_), and mean weighted skin (*T*_sk_, 10 sites) temperatures were recorded continuously. The trials were terminated if the rectal temperature dropped below 35°C or finger temperature dropped below 5°C

In study 2, eight volunteers sat quietly at an ambient temperature of 0.48 ± 0.53°C and RH 51 ± 3% with a wind velocity of 1.34 m·s^−1^ for 120 min (Castellani et al. 2018). They wore 3 layers of the ECWCS on the torso (silk weight underwear, fleece mid-layer, and soft shell outer layer) and 2 layers on the legs (silk weight underwear and soft shell outer layer) as well as wool socks, the Army Improved Intermediate Cold-Wet Boot, and an Army fleece hat but were bare handed throughout the exposures. Skin temperature and heat flux (HF) of the volunteers’ non-dominant side were measured at 13 sites (e.g., finger and toe) every minute throughout the baseline and cold experimental time periods. An 11-site formula was used for calculating mean weighted *T*_sk_.

In study 3, four volunteers wore the ECWCS during 120-min exposure to a −40°C environmental condition (Hickey Jr et al. 1993). The ECWCS consisted of undershirt/underpants, overalls/jacket, batting field coat liner/trouser liner, all weather rain trouser/parka with hood, balaclava (worn under parka hood), wool trigger finger mitten insert, extreme cold weather mitten set, cushion sole sock, and white vapor barrier boots. Volunteers sat quietly for 25 min, followed by 5 min of treadmill walking during each 30-min time period. Rectal temperature and skin temperatures at the finger, toe, and scapula were measured and recorded at 5-min intervals. One of the criteria for termination of an exposure session was a skin temperature that fell below 10°C.

### Comparison

**Fig. 8:**
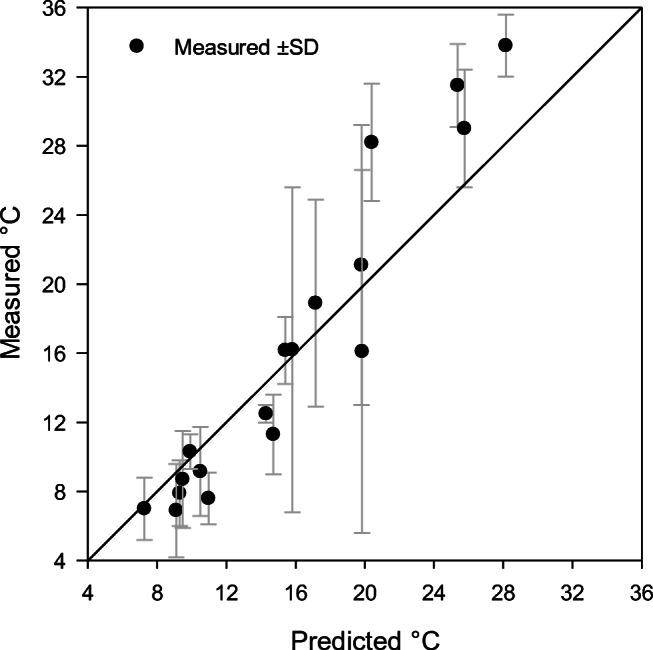
Comparison of measured and CoWEDA-predicted finger temperatures at the end of rest and exercise at 0, −20, −30, and −40°C wearing Extended Cold Weather Clothing System (ECWCS) and different gloves and boots

**Fig. 9:**
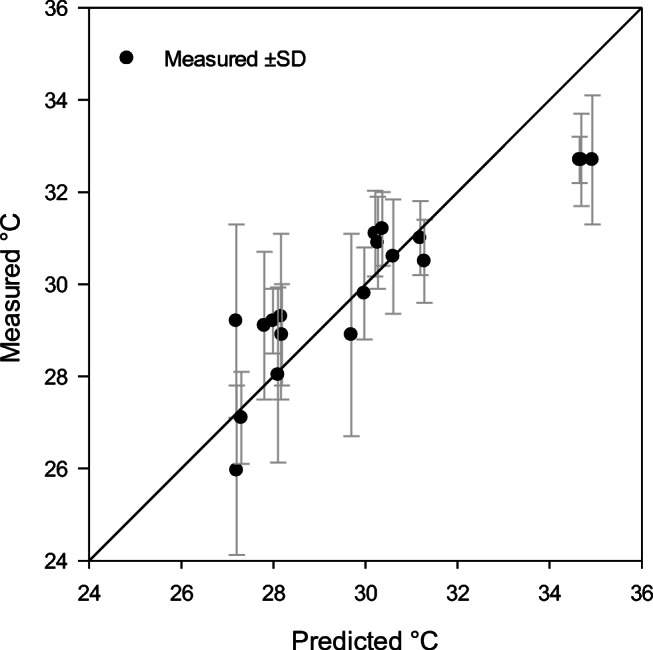
Comparison of measured and CoWEDA-predicted mean skin temperatures at the end of rest and exercise at 0, −20, −30, and −40°C wearing Extended Cold Weather Clothing System (ECWCS) and different gloves and boots

**Fig. 10:**
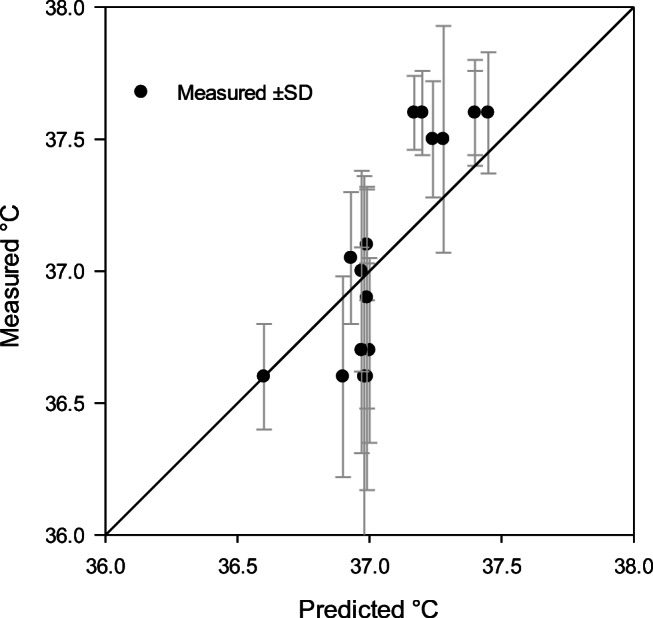
Comparison of measured and CoWEDA-predicted core temperatures at the end of rest and exercise at 0, −20, −30, and −40°C wearing Extended Cold Weather Clothing System (ECWCS) and different gloves and boots

The measured skin, finger, and core temperatures at the end of each cold exposure, ranging from 37 to 120 min, were compared with skin and finger temperatures predicted by CoWEDA. Figure 8 shows the measured and predicted finger temperatures and reveals that twelve of eighteen predicted finger temperatures were within the range of measured values ± SD. Figure 9 summarizes the measured and predicted skin temperatures and indicates that fourteen of eighteen predicted skin temperatures were within the range of measured ± SD. Figure 10 shows the measured and predicted core temperatures and reveals that thirteen of seventeen predicted core temperatures were within the range of measured values ± SD. Furthermore, differences between observed temperatures and CoWEDA predictions were evaluated by comparing the root mean square deviation (RMSD) with the observed standard deviation. The RMSD for the finger, mean skin, and core temperatures were 3.30, 1.18, and 0.26°C, respectively, while the corresponding average SD of the observed values were 3.56, 1.22, and 0.31°C separately. Thus, the validation shows that the CoWEDA prediction is acceptable.

**Fig. 11:**
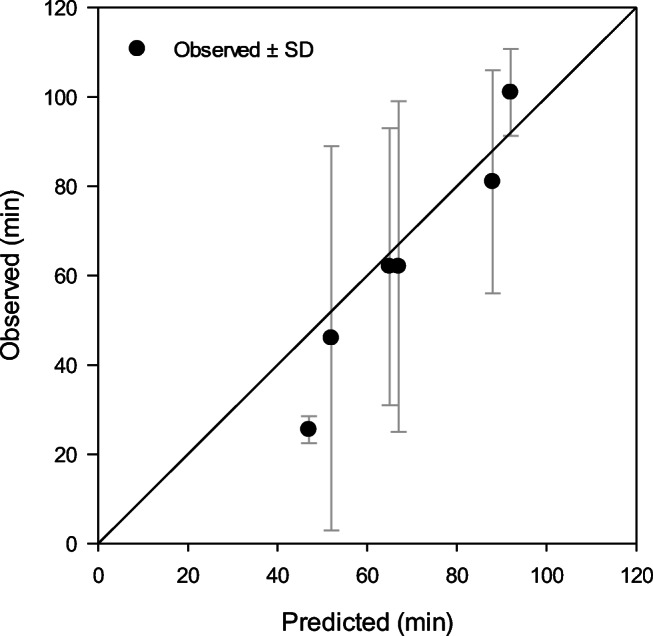
Comparison of measured and CoWEDA-predicted finger endurance times during rest at −20, −30, and −40°C wearing three different gloves and Extended Cold Weather Clothing System (ECWCS) and different gloves and boots

During exposures to extreme cold conditions of −20°C to −40°C, six trials at rest were terminated earlier than the exposure limit of 2 h, mainly due to low finger temperatures (Gonzalez et al. 1989). Thus CoWEDA was used to predict endurance time which was the duration when the finger temperatures reached the 5°C values at termination. Figure 11 shows the observed and predicted endurance times. The predicted endurance times agree with the observed value and were within the observed endurance time ± SD except for one condition.

## Discussion

CoWEDA is a knowledge-based decision aid developed for a variety of users, such as soldiers and outdoor workers, to guide selection of the most appropriate cold weather ensemble(s) relative to anticipated activities and environmental conditions. CoWEDA integrates human-centric performance metrics of cold weather ensembles, a thermoregulatory model (SCTM), and a database of clothing biophysical properties into a single user-friendly software application. It is the first tool that allows users to build their own ensembles from the inventory and interpret the selection using physiological terms and consequences. CoWEDA focuses on users’ physiological status and safety, and its outcomes are easy to understand and use. Comparison with measured skin temperatures during exposure to 0 to −40°C environments shows that the accuracy of CoWEDA prediction is acceptable. CoWEDA is a state-of-the-art tool for cold activity planning, clothing selection, risk assessment, and cold injury prevention.

CoWEDA is human centric and aims to ensure operation safety and optimal human performance. Cold stress degrades physical and mental performance, and cold injuries can be life-changing or even life-threatening. Selecting appropriate clothing ensembles is one of the most important measures to counter cold stress and prevent cold injury. The performance metrics used in CoWEDA provides quantitative measures to assess the protective role an ensemble can provide. The endurance times illustrate the protection level or effectiveness of the selected ensemble. ISO 11079 predicts insulation values that are required to maintain body heat balance as well as duration limited exposure and a required recovery time, but do not predict insulation values required to prevent extremity cold injury or local cooling (http://www.design.lth.se/english/the-department/research-laboratories/climate-lab/tools/calculations-for-ireq-and-wct/, accessed on 2/22/2021). Extremity cold injury is very common in field and practical settings, such as rescue operations (Kupper et al. 2003) and exercise or military operations in cold (Sullivan-Kwantes et al. 2017; Sullivan-Kwantes and Goodman 2017; O'Donnell et al. 2017). Extremity protection is a critical part of the overall cold injury prevention strategy. The newly developed performance metrics for cold weather ensembles are more comprehensive than both the existing standards and address the complex requirements of cold weather ensembles: adequate protection to prevent both frostbite and hypothermia while avoiding excessive sweating. CoWEDA outcomes are directly related to physiological status, are easy to understand, and can be directly incorporated into mission planning and risk assessments.

CoWEDA is the first tool that allows users to select from an inventory of available individual clothing items and accessories to build an ensemble suitable for the environmental conditions and cold weather activity. Insulation values of specific ensembles and some individual clothing items are available in clothing databases in the literature (ISO 11079 2007; ASHRAE 2013; McCullough et al. 1985; McCullough et al. 1989). Guidance for cold stress and injury management usually includes tables or charts for clothing insulation requirements relative to metabolic rates and environmental conditions (Castellani et al. 2006; Department of the Army 2005). Due to the lack of standard procedures or calculation methods, it is difficult to use insulation values of individual items to calculate the total insulation of an ensemble, and it is also difficult to build or select an ensemble that provides an insulation value that is recommended by the guidance or standards. These limitations present challenges even for practitioners, e.g., industrial hygienists and occupational safety/health experts, trying to use the IREQ model in ISO 11079 to assess cold environment risk (d'Ambrosio Alfano et al. 2013). A recent survey revealed that when Arctic open-pit miners selected their own clothing, they took into account task duration, environmental condition, experience, and wetness due to sweating (Jussila et al. 2017). CoWEDA provides science-based guidance that can reinforce the user’s confidence in their clothing choices or, if necessary, provide a check against possible over-confidence. Importantly, CoWEDA directly relates a selected ensemble or clothing items to physiological consequences and can assist end users, such as the open-pit miners, in selecting clothing most suitable to their needs. CoWEDA can be used not only by professionals such as occupational ergonomists or hygienists but also by end users.

CoWEDA is an interactive supplement to current guidance for cold stress and injury management. The current guidance usually relies on wind chill temperature (WCT) for frostbite risk assessment (https://www.weather.gov/oun/safety-winter-windchill, accessed 10/17/2019), with the risk of frostbite presented as a look-up table. Frostbite times of exposed cheek skin in the WCT tables are a function of ambient temperature and air velocity and are limited to four time categories: unlimited, 30 min, 10 min, and 5 min. The endurance time of exposed skin, one of the CoWEDA outputs, captures basically the same information but presents it as a value rather than as a look-up table. This exposure time is a function of ambient temperature, air velocity, ensemble worn, and activity and incorporates more of the factors that actually determine the safe exposure time**.** For example, in air temperatures of −32°C with a 2.2 m·s^−1^ air velocity, frostbite time is calculated at about 30 min according to the WCT table. CoWEDA predicts the endurance time of exposed skin to be about 18, 24, and 36 min when a mild, moderate, or extreme cold weather gear (a preset ensemble) is worn. Thus, the CoWEDA output for exposed skin potentially is a significant expansion of the WCT-based guidance as it takes into account air temperature, wind speed, activity level, and clothing. Perhaps of equal or greater importance, CoWEDA provides an assessment of the risk of hypothermia, as no currently provided guidance directly relates WCT to the risk of hypothermia. Improved awareness is a critical element of cold injury prevention (Imray and Oakley 2005; Makinen and Hassi 2009). CoWEDA is an interactive tool that translates weather forecast data into appropriate clothing items and physiological consequences, educates users about the dangers of cold weather, and improves their awareness in such environments.

The CoWEDA architecture is designed in such a way that it can be easily adapted to create variations for different user communities, such as outdoor utility workers and outdoor recreational participants. The adaption requires revision of the physiological criteria and establishment of the clothing database. The physiological criteria reflects the operation requirements, and the numbers in Table 1 are currently set to prevent cold injury. If the purpose is to ensure manual performance to complete certain tasks, a skin temperature of ~13–16°C would be appropriate (Geng 2001; Heus et al. 1995; Xu et al. 2005a). The database currently includes items of the US Army cold weather ensemble, and these items can be replaced or supplemented by new items to create a database for specific user communities. CoWEDA variations can help widen the scope of users to select ensembles and manage their working schedule in a rational manner.

The limitation of this study is the small sample sizes of validation data, which was collected at a low wind speed and low work intensity. The performance of CoWEDA will be further evaluated in future research when more human data becomes available, especially data with a wide range of environmental conditions and work intensities. At this point, the CoWEDA is intended for general guidance, not for individualized guidance. In future, we hope that CoWEDA will be able to predict the probability of cold injuries and to identify “who” are more likely to have cold injury.

## Conclusions

The Cold Weather Ensemble Decision Aid (CoWEDA) was developed to guide end users to select the most appropriate cold weather clothing relative to the anticipated activities and environmental conditions. CoWEDA focuses on physiological status and safety of users rather than the biophysical properties of clothing. The newly defined performance metrics of cold weather clothing aims to not only prevent frostbite and hypothermia but also avoids excessive sweating. CoWEDA is the first tool that allows users to build their own ensembles from the available clothing items by body regions and to interpret the selection using physiological terms and consequences. Validation with observed physiology data shows that the accuracy of CoWEDA prediction is acceptable during exposure to 0 to −40°C environments. CoWEDA represents a significant improvement over current existing standards or guidance for cold weather clothing selection and a significant expansion of WCT-based guidance for cold injury prevention and mission planning.

## Declarations

### Disclaimer

Approved for public release; distribution is unlimited. The opinions or assertions contained herein are the private views of the author(s) and are not to be construed as official or reflecting the views of the Army or the Department of Defense. Any citations of commercial organizations and trade names in this report do not constitute an official Department of the Army endorsement of approval of the products or services of these organizations.

## References

[CR1] Aptel M (1988). Comparison between required clothing insulation and that actually worn by workers exposed to artificial cold. Appl Ergon.

[CR2] ASHRAE (2013) Thermal Comfort. In: 2013 ASHRAE Handbood Fundamentals. American Society of Heating, Refrigerating and Air Conditioning Engineers, Atlanta, pp 9.1-9.32

[CR3] ASTM International (2016a) Standard practice for determining the temperature ratings for cold weather protective clothing. In. West Conshohocken, PA

[CR4] ASTM International (2016b) Standard test method for measuring the evaporative resistance of clothing using a sweating manikin (ASTM F2370). In. West Conshohocken, PA

[CR5] ASTM International (2016c) Standard test method for measuring the thermal insulation of clothing using a heated manikin (ASTM F1291). In. West Conshohocken, PA

[CR6] Besnard Y, Launay JC, Guinet-Lebreton A, Savourey G (2004). PREDICTOL: a computer program to determine the thermophysiological duration limited exposures in various climatic conditions. Comput Methods Prog Biomed.

[CR7] Castellani JW, Tipton MJ (2015). Cold stress effects on exposure tolerance and exercise performance. Compr Physiol.

[CR8] Castellani JW, Young AJ, Ducharme MB, Giesbrecht GG, Glickman E, Sallis RE (2006). Prevention of Cold Injuries during Exercise. Med Sci Sports Exerc.

[CR9] Castellani JW, O'Brien C, Tikuisis P, Sils IV, Xu X (2007). Evaluation of two cold thermoregulatory models for prediction of core temperature during exercise in cold water. J Appl Physiol.

[CR10] Castellani JW, Yurkevicius BR, Jones ML, Driscoll TJ, Cowell CM, Smith L, Xu X, O'Brien C (2018) The effect of localized microclimate heating on peripheral skin temperatures and manual dexterity during cold exposure. J Appl Physiol (Bethesda, Md : 1985). doi:10.1152/japplphysiol.00513.2018 10.1152/japplphysiol.00513.2018 30138077

[CR11] d'Ambrosio Alfano FR, Palella BI, Riccio G (2013). Notes on the implementation of the IREQ model for the assessment of extreme cold environments. Ergonomics.

[CR12] DeGroot DW, Castellani JW, Williams JO, Amoroso PJ (2003). Epidemiology of U.S. Army cold weather injuries, 1980-1999. Aviat Space Environ Med.

[CR13] Department of the Army (2005) Prevention and management of cold-weather injuries. Technical Bulletin Medicine MED 508, Washington, DC: Headquaters

[CR14] Gao C, Lin LY, Halder A, Kuklane K, Holmer I (2015). Validation of standard ASTM F2732 and comparison with ISO 11079 with respect to comfort temperature ratings for cold protective clothing. Appl Ergon.

[CR15] Geng Q (2001) Hand cooling, protection and performance in cold environments. Luleå University of Technology, National Institute for Working Life

[CR16] Gonzalez RR, Endrusick TL, Santee WR (1989) Thermoregulatory responses in the cold: eof an extended cold weather clothing system (ECWCS). In, 1989. 15th Commonwealth Defence Conference on Operational Clothing and Combat Equipment. Ottawa, Canada

[CR17] Gonzalez R, McLellan TM, Withey WR, Chang SK, Pandolf KB (1997). Heat strain models applicable for protective clothing systems: comparison of core temperature response. J Appl Physiol.

[CR18] Gonzalez RR, Endrusick TL, Santee WR (1998). Thermoregulatory responses to cold: effects of handwear with multi-layered clothing. Aviat Space Environ Med.

[CR19] Haslam RA, Parsons KC (1994). Using computer-based models for predicting human thermal responses to hot and cold environments. Ergonomics.

[CR20] Heil K, Thomas R, Robertson G, Porter A, Milner R, Wood A (2016). Freezing and non-freezing cold weather injuries: a systematic review. Br Med Bull.

[CR21] Heus R, Daanen HAM, Havenith G (1995). Physiological criteria for functioning of hands in the cold: a review. Appl Ergon.

[CR22] Hickey Jr CA, Woodward Jr AA, Hanlon WE (1993) A pilot study to determine the thermal protective capability of electrically heated clothing and boot inserts. ARMY RESEARCH LAB ABERDEEN PROVING GROUND MD

[CR23] Holmer I (1984). Required clothing insulation (IREQ) as an analytical index of cold stress. ASHRAE Trans.

[CR24] Holmer I (1988). Assessment of cold environments in terms of required insulation. Arctic Med Res.

[CR25] Holmer I (2009). Evaluation of cold workplaces: an overview of standards for assessment of cold stress. Ind Health.

[CR26] Imray C, Oakley E (2005). Cold still kills: cold-related illnesses in military practice freezing and non-freezing cold injury. J R Army Med Corps.

[CR27] ISO 11079 (2007) Ergonomics of the thermal environment -- determination and interpretation of cold stress when using required clothing insulation (IREQ) and the assessment of local cooling effects. Geneva

[CR28] Jussila K, Rissanen S, Aminoff A, Wahlstrom J, Vaktskjold A, Talykova L, Remes J, Manttari S, Rintamaki H (2017). Thermal comfort sustained by cold protective clothing in Arctic open-pit mining-a thermal manikin and questionnaire study. Ind Health.

[CR29] Kuklane K (2009). Protection of feet in cold exposure. Ind Health.

[CR30] Kupper T, Steffgen J, Jansing P (2003). Cold exposure during helicopter rescue operations in the Western Alps. Ann Occup Hyg.

[CR31] Makinen TM, Hassi J (2009). Health problems in cold work. Ind Health.

[CR32] McCullough EA, Jones BW, Huck J (1985). A comprehensive data base for estimating clothing insulation. ASHRAE Trans.

[CR33] McCullough EA, Jones BW, Tamura P (1989). A database for determining the evaporative resistance of clothing. ASHRAE Trans.

[CR34] O'Donnell FL, Stahlman S, Oetting AA (2017) Update: Cold weather injuries, active and reserve components, U.S. Armed Forces, July 2012-June 2017. Msmr 24 (10):12-21 29077423

[CR35] Potter AW, Gonzalez JA, Carter AJ, Looney DP, Rioux TP, Srinivasan S, Sullivan-Kwantes W, Xu X (2018) Comparison of cold weather clothing biophysical properties: US Army, Canadian Department of National Defence, and Norwegian Military. USARIEM Report T18-02; AD#1051229, Natick United States,

[CR36] Rioux T, Karis A, Moore B, Xu X (2018) Biophysical evaluation of individual component levels and selected configurations of the United States Marine Corps Cold-Weather Clothing Ensemble. USARIEM Technical Note T 18-02, Natick, MA

[CR37] Rioux T, Gonzalez JA, Karis A, Potter AW, Xu X (2020) Biophysical properties of five cold weather clothing systems and the predicted regional properties of ensembles. USARIEM Technical Report T 21-03. AD1115339, Natick, MA

[CR38] Sullivan-Kwantes W, Goodman L (2017). The new cold war. Temperature (Austin, Tex).

[CR39] Sullivan-Kwantes W, Dhillon P, Goodman L, Knapik JJ (2017). Medical encounters during a joint Canadian/U.S. exercise in the high Arctic (exercise Arctic Ram). Mil Med.

[CR40] Xu X, Santee WR (2011). Sweat loss prediction using a multi-model approach. Int J Biometeorol.

[CR41] Xu X, Werner J (1997). A dynamic model of the human/clothing/environment-system. Appl Hum Sci.

[CR42] Xu X, Santee W, Giesbrecht G, Gonzalez R (2005). Prediction of hand manual performance during cold exposure. SAE 2004. Trans J Aerosp.

[CR43] Xu X, Tikuisis P, Gonzalez R, Giesbrecht G (2005). Thermoregulatory model for prediction of long-term cold exposure. Comput Biol Med.

[CR44] Xu X, Castellani JW, Santee W, Kolka M (2007) Thermal responses for men with different fat compositions during immersion in cold water at two depths: prediction versus observation. Eur J Appl Physiol 100, 79(1):–88. 10.1007/s00421-007-0393-z 10.1007/s00421-007-0393-z 17508227

[CR45] Xu X, Turner CA, Santee WR (2011). Survival time prediction in marine environments. J Therm Biol.

